# The impact of patient compliance with adjuvant radiotherapy: a comprehensive cohort study

**DOI:** 10.1002/cam4.114

**Published:** 2013-08-20

**Authors:** Harun Badakhshi, Arne Gruen, Jalid Sehouli, Volker Budach, Dirk Boehmer

**Affiliations:** 1 Department of Radiation Oncology, Charité School of Medicine and University HospitalBerlin, Germany; 2 Department of Gynaecology, Charité School of Medicine and University HospitalBerlin, Germany

**Keywords:** Adherence, breast cancer, compliance, quality of care, radiotherapy

## Abstract

Postoperative radiotherapy (RT) is the standard of care for early stage breast cancer. It reduces the risk for local recurrence and prolongs survival. We assessed whether, the omission of RT because of patient's preference may influence the prognosis and, thus, the quality of cancer care. Detailed information from a prospectively collected database of a breast cancer center was analyzed. Multiple regression analysis and univariate and multivariate analysis for risk factors for recurrence were performed. The entire cohort of primary breast cancer patients in a given time period was analyzed. Data from 1903 patients undergoing treatment at breast cancer center between 2003 and 2008 were used. All patient underwent breast conserving surgery and RT was performed for all patients of the cohort. Local tumor control and disease-free survival were calculated. After a median follow-up of 2.18 years (maximum 6.39 years), 5.5% of patients did not follow guideline-based recommendations for RT. There was a significant correlation between noncompliance and patient's age, adjuvant hormonal therapy (97.0%), and adjuvant chemotherapy (96.8%). Seventy local recurrences occurred that corresponds to a local recurrence rate of 3.9%. The difference in regard to local recurrence-free 5-year survival between the compliant patients and the noncompliant patients is absolute 17.9 (93.3% and 75.4%). Noncompliant patients had suffered a 5.02-fold increased risk of local recurrence than compliant patients. The omission of RT after breast-conserving surgery results in a higher local failure rate and significantly worsens clinical outcome. Age may play an important role because of the comorbidities of aged patients or the assumed low RT tolerance in this group. On a clinical level, this data suggests that improvement is needed to correct this situation, and the question remains as to how best to improve RT compliance.

## Introduction

In Western countries, one of every eight women is at risk for breast cancer. In most patients in whom breast cancer is detected early, breast-conserving surgery with postoperative radiotherapy (RT) is the primary therapeutic strategy. RT reduces the rate of local relapse and improves long-term survival^1^. The standard RT regimen lasts for approximately 6 weeks (five applications per week) and involves a substantial costs and allocation of human and structural resources within the health care system. In order to achieve the best therapeutic ratio, it is important for patients and caregivers to adhere to a postoperative RT course that is based upon national guidelines and the best available evidence^2^. However, in daily clinical practice, not every patient with an early diagnosis of breast cancer is treated according to evidence-based guidelines, and noncompliance may be caused by various factors. For example, a lack of adequate information on the rationale for receiving RT, unconvincing biological and clinical explanations, and a diffuse fear of radiation in general may generate reluctance^3^,^4^.

In the context of health care research, the quality of care given to specific groups of patients can be adequately judged by prospective clinical collection of data and analyses of clinical outcomes on an individual basis^5^–^7^. These results might enable the detection of problems concerning treatment quality, such as over or underuse of suggested options, which may be linked to the adherence of the attending physician to the suggested guidelines. Determination and reformation of these problems could improve the quality of care given to patients, improve the decision-making process, and allow the efficient allocation of human and structural resources^8^–^12^.

The aim of this study was to investigate patient compliance with RT after breast-conserving surgery through a population-based investigation of a large cohort in terms of actual clinical outcomes. This study was not intended to be a phase IV trial to confirm well-known level I data on the management of early breast cancer but was intended to analyze the outcomes of routine patient care from the patient's perspective. We have therefore rated local control and survival as the most relevant clinical outcomes that may be clearly influenced by the application or omission of RT and investigated the quantity of cases receiving no RT, and, then the causality in regard to themes of compliance.

## Methods

Included in this study were 1903 patients who underwent treatment for nonmetastatic early breast cancer or ductal carcinoma in situ (DCIS) in the breast at the certified Interdisciplinary Breast Centre between 1 November 2003 and 31 December 2008. During this time period, 2041 operations for primary breast cancer or DCIS were performed, which includes multiple procedures for 68 patients who presented with bilateral (synchronous or asynchronous) lesions and, therefore, underwent two separate operations. Patients with malignant phyllodes tumor, sarcoma, or metastatic disease were excluded from this study. The date of core biopsy was considered as the date of tumor diagnosis.

Medical records of the included patients were evaluated. One hundred and six (5.6%) patients were lost to follow-up, and thus, 1797 patient records were included in the final analysis. The prospective recording, storage, distribution, and compilation of all primary data, including histology, radiological findings, surgery, and pathology reports, the decisions of the interdisciplinary tumor conferences, all relevant details of delivered treatments, and information pertaining to follow-up visits in the outpatient clinics, were performed using a hospital-based oncology-dedicated software (ODSeasy, Asthenis Ltd., Aschheim, Germany). The study center gathered secondary data by collecting and evaluating reports and patient records from other institutions, including gynecology, radiology, and RT facilities. After approval was obtained from the institutional review board, a questionnaire was sent to those patients whose last follow-up visit was more than 6 months ago. In cases of no reply to the questionnaire, we performed a structured interview via telephone call. The questionnaire contained five questions about the patient's general medical condition, recent serious medical events, date and findings of the most recent breast imaging study with regard to local recurrence, treatment at other institutions, and severe treatment-related toxicity. Statistical analysis was performed using SPSS software (SPSS 16; New York, NY). The current study was an exploratory approach to determine the actual clinical outcomes with regard to patient compliance RT and did not aim to prove the superiority or inferiority of certain procedures. Therefore, we did not apply the Bonferroni adjustment to the *P*-values in our statistical tests. Statistical significance was accepted at a significance level of *P *≤ 0.05. Descriptive data analysis was performed using conventional position calculation (mean, median, minimum, and maximum) and variability (standard deviation) for quantitative variables and absolute and relative frequencies for categorical data.

## Results

The mean age of the patients was 59.9 years (median, 62; range 23–96), and the median observation period was 2.1 years (maximum, 6.39 years). Tables 1 and 2 summarize the general histopathological and immunohistochemical characteristics of the cohort, respectively. RT noncompliance varied between 3.6% and 5.8%. Overall, 104 patients did not follow the guideline-based recommendations for RT. Noncompliance was associated with patient age (*P* < 0.0005; Table 3). Compliance with RT was statistically higher in patients who received adjuvant hormone therapy (97.0%) than in patients who did not receive hormone therapy (93.1%) (*P *< 0.0005) and in patients who received adjuvant chemotherapy (96.8%) than in patients who did not receive chemotherapy (93.9%) (*P* = 0.027). Patients with larger tumors (pT3/4 tumors) were also less compliant with RT (*P *= 0.024). No association (by the chi-square test) could be found with respect to tumor stage (*P *= 0.177), nodal status (*P *= 0.466), tumor grade (*P *= 0.063), or the application of neoadjuvant chemotherapy (*P *= 0.374). In multiple logistic regression analyses for those factors that had a significant impact on the omission of RT in univariate analyses, only age and hormone therapy were found to have a significant correlation with the frequency of nonimplementation of RT (Table 4).

**Table:** Pathohistological data of the entire cohort

Measured parameter	Number	%
Resection margin		
R0	1753	97.8%
R1	36	2%
Rx	3	0.2%
*T* status		
Intra situ	228	12.7%
1	838	46.8%
2	580	32.4%
3	97	5.4%
4	49	2.7%
*N* status		
Negative	1124	62.7%
1–3 positive LN	353	19.7%
4–9 positive LN	124	6.9%
>10 positive LN	80	4.5%
NN	111	6.2%
Lymphatic vessel invasion		
Negative	955	53.5%
Positive	310	17.3%
Vessel invasion		
Negative	803	44.8%
Positive	26	1.5%
Grading		
G1	328 for IDC	21%
	32 for DCIS	14%
G2	830 for IDC	53.1%
	37 for DCIS	16.2%
G3	406 for IDC	25.9%
	93 for DCIS	40.8%
G X	0 for IDC	0
	66 for DCIS	29%

**Table:** Immunohistochemistry (ER, PR) and FISH (Her2 neu) data of the entire cohort

Measured parameter	Number	Percentage
Estrogen receptors		
Negative	262 for IDC	16.8%
	36 for DCIS	15.8%
Positive	1298 for IDC	83%
	130 for DCIS	57%
Unknown receptor status	4 for IDC	0.2%
	62 for DCIS	27.2%
Progesterone receptors		
Negative	477 for IDC	30.5%
	61 for DCIS	26.7%
Positive	1081 for IDC	69.1%
	106 for DCIS	46.5%
Unknown receptor status	6 for IDC	0.4%
	61 for DCIS	26.4%
Her2 neu amplification		
Negative	1246 for IDC	79.7%
	32 for DCIS	14%
Positive	300 for IDC	19.2%
	21 for DCIS	9.2%
Unknown status	18 for IDC	1.1%
	175 for DCIS	76.8%

**Table:** Compliance with the recommendation for adjuvant radiotherapy

		With RT	NO RT	Total
Recommendation	Yes	1346 (93.4%)	95 (6.6%)	1441 (100%)
	Ratio to all recommendations	70.7%	5 %	75.7%
	No	9 (1.9%)	453 (98.1%)	462 (100%)
	Ratio to all recommendations	0.5%	23.8%	24.3%
Total		1355	548	1903
	Ratio to all recommendations	71.2%	28.8%	100%

**Table:** Multiple regression analysis for factors of noncompliance

Measured parameter	Noncompliance	OR	95% confidence interval	*P*-value
Age	5.5 (1903)	1.04	Minimum 1.02, maximum 1.05	<0.0005
Hormonal therapy	Yes: 3.0 (696)	1	Minimum 1.49, maximum 3.98	<0.0005
	No: 6.9 (1207)	2.44		
Chemotherapy	Yes: 3.2 (402)	1	Minimum 0.75, maximum 2.56	0.301
	No: 6.1 (1501)	1.38		
T status	Is: 6.0 (228)	1	Minimum 0.43, maximum 1.56	0.219
	1: 4.2 (838)	0.82	Minimum 0.62, maximum 2.27	0.543
	2: 6.3 (580)	1.19	Minimum 1.71, maximum 4.17	0.604
	3: 8 (97)	1.71	Minimum 1.75, maximum 4.99	0.241
	4: 11.5 (402)	1.75		0.301

We attempted to determine what caused the reluctance in patients who did not receive RT. A vast majority of these patients (80%) were not convinced, after the first postoperative clinical visit, that they would need RT. In 55 out of the 104 cases (52.9%) of nonadherence to RT recommendations, patients claimed that RT would have been unnecessary, too dangerous (due to radiation), or too stressful (due to the logistics of treatment, proximity of living area to the treatment center, and other chronic health issues). For the remaining 49 patients, the reasoning for omission of RT given during their interviews did not help to understand the cause of RT rejection.

During the observation period (date of operation to 30 June 2010), 70 local recurrences (local recurrence rate of 3.9%) occurred in the overall population. The local recurrence-free 5-year survival rate of all patients was 92.5%. The difference in the 5-year local recurrence-free survival rate between the RT compliant and noncompliant patients was 17.9% (93.3% and 75.4%, respectively). Potential cofactors for this difference are shown in Table 5 (univariate testing) and Table 6 (multivariate testing). With a significance level of 5% in univariate Cox regression, a differential risk was determined to exist between the two groups of patients. With regard to the incidence of local recurrence, the hazard ratio (HR) was 4.056 for omission of RT (*P* < 0.0005; 95% confidence interval [CI] 2.13–7.72). Noncompliant patients presented with a 5.02-fold increased risk in local recurrence when compared to RT-compliant patients. This is likely attributable to not only the known predictors determined in multivariate analysis, but also the lack of compliance with recommendations by the attending physician.

**Table:** Univariate analysis of factors with a significant influence on recurrence risk

Measured parameter	Recurrence percentage (*n*)	HR	95% confidence interval	*P-*value
Compliance	Yes: 3.4(1711)	1	Minimum 2.13, maximum 7.72	<0.0005
	No: 12.8 (86)	4.06		
Grading	G1: 1.4 (351)	1		<0.0005
	G2: 2.9 (854)	2.07	Minimum 2.07, maximum 5.41	0.137
	G3: 6.9 (495)	5.46	Minimum 2.13, maximum 13.9	<0.0005
Hormone receptors	Yes: 2.8 (1434)	1		<0.0005
	No: 8.2 (294)	3.07	Minimum 1.85, maximum 5081	

**Table:** Multivariate analysis of factors with a significant influence on recurrence risk

Measured parameter	Recurrence percentage (*n*)	HR	95% confidence interval	*P-*value
Compliance	Yes: 3.4 (1711)	1	Minimum 2.50, maximum 10.05	<0.0005
	No: 12.8 (86)	5.02		
Grading	G1: 1.4 (351)	1		<0.0005
	G2: 2.9 (854)	2.22	Minimum 0.77, maximum 6.46	0.142
	G3: 6.9 (495)	5.67	Minimum 2.37, maximum 19.24	<0.0005

## Discussion

The principal finding of this study was that the omission of RT after breast-conserving surgery in patients with early breast cancer leads to a clinically significant deterioration in local tumor control, thereby negatively affecting the prognosis of these patients.

**Figure 1:**
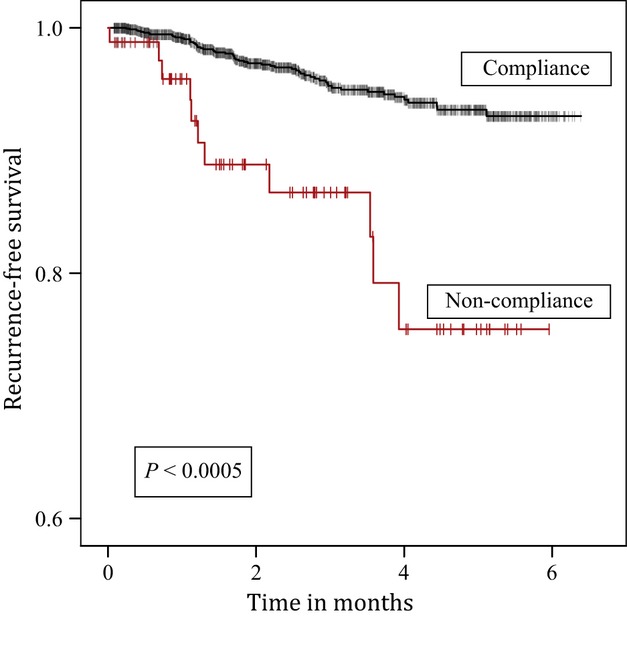
Recurrence free survival in regard to compliance to radiotherapy.

Adherence of physicians with evidence-based guidelines and compliance of patients to the recommended therapy are aspects of quality of care and good patient-oriented practice. This relates to objective and measurable clinical outcomes, especially in the realm of clinical oncology.

In the present study, the difference in the local recurrence-free survival rate between compliant and noncompliant patients at 5 years was 17.9%. This is a clear indication that omission of RT after breast-conserving surgery leads to a worse outcome. We studied the correlation between noncompliance and patient and tumor variables. In 48.1% of noncompliant patients, the reason for RT noncompliance could not be established: patients either did not answer the questionnaire sent to them or refused to explain their motivation. The data from the present study is concordant with the Early Breast Cancer Trialists' Collaborative Group (EBCTCG) results, which showed a high level of evidence indicating that patients not receiving RT for early breast cancer have a higher risk of local tumor relapse and may have negative consequences with regard to survival. The EBCTCG analysis also showed a correlation between local tumor control and survival. Approximately one breast cancer death was avoided by year 15 for every four recurrences avoided by year 10. This 1:4 ratio should be considered in patient discussions concerning RT^1^.

The direct and statistically significant relationship between age and RT compliance for early breast cancer calculated in the present analysis is worthy of consideration. Older patients received proportionately less RT than younger patients. A Dutch population-based study showed that, of 5577 patients receiving breast-conserving surgery, 96.5% received RT. Withholding RT after breast-conserving surgery was associated with age. Whereas 97.7% of the patients <70 years received RT, the percentages of patients aged 70–74, 75–79, and ≥80 years who received RT were 95.8, 90.9, and 57.4%, respectively (*P *< 0.001)^13^. Similar results according to age were also seen in another study^14^. Omission of RT significantly decreased overall survival (≤69 years, HR = 3.29, *P* < 0.0001; ≤70 years, HR = 1.89, *P* = 0.0005) and disease-free survival (≤69 years, HR = 3.45, *P *< 0.0001; ≥70 years, HR = 2.14; *P *< 0.0001), indicating that a deviation from the standard treatment concept results in a poor outcome^14^. One possible explanation for age being a factor in RT noncompliance might be the temporal changes in patterns and standards of treatment.

Increase in and systematic administration of endocrine therapy may compensate for the omission of RT. The US-based CALGB study, comparing lumpectomy plus tamoxifen with and without radiation in breast cancer patients older than 70 years, found only a small, insignificant excess risk of local recurrence in the nonirradiated group and no differences in risk or survival in cases of distant metastases^15^. The age of the patient might be the missing link between the context of patient compliance, consequences, and the quality of the doctor's informed consent discussion with the patient and the clinical outcome and quality of care. There is certainly a need for a specific focus on elderly female patients with early breast cancer in order to determine their interaction with caregivers and compliance with recommended treatment models^16^–^18^.

Issues related to compliance with a recommended treatment or long-term adherence to medication have been widely discussed, with special regard to clinical outcome and quality of care. There is certainly a burden not just for the patients but also for caregivers and the healthcare system. These costs are both personal and societal, such as those caused by complications and hospitalization^19^,^20^.

With regard to RT compliance, there is a lack of valid patient data. A literature search on PubMed for publications concerning RT compliance, with advanced settings of both terms in the title of the paper, articles written in English, and articles published in the last 5 years, revealed just 13 papers, of which only two concerned RT compliance in breast cancer^21^,^22^.

The main strength of this analysis was the inclusion of the entire cohort of treated patients with nonmetastatic early breast cancer from the setup of the Breast Care Centre in 2003 until 2008. This population-based investigation and its data analysis were performed in terms of real clinical outcomes in a nonselective and comprehensive number of patients under the conditions of routine care and outside of the rigid grid of a randomized controlled trial.

The major weaknesses of this cohort study are the short median follow-up time of 2.18 years and, more importantly, the inability to find an objective basis for noncompliance with RT. Not being able to reach patients during follow-up in order to understand their preferences is regarded as a methodological weakness. The main issue for physicians was the inability to convince the patient during their informed consent discussions that RT was necessary.

This study showed that the omission of RT after breast-conserving surgery results in a higher local failure rate and significantly worsens clinical outcome. Age may play an important role because of the comorbidities of aged patients or the assumed low RT tolerance in this group. On a clinical level, this data suggests that improvement is needed to correct this situation, and the question remains as to how best to improve RT compliance.

Efficient and feasible methods of training for oncology caregivers, with regard to conducting informed consent discussions in an adequate manner according to the patient's educational, cognitive, social, and current emotional condition, is challenging, but necessary, to ensure patient compliance with suggested therapies.

## Conclusion

The omission of RT after breast-conserving surgery results in a higher local failure rate and significantly worsens clinical outcome. Age may play an important role because of the comorbidities of aged patients or the assumed low RT tolerance in this group. On a clinical level, this data suggests that improvement is needed to correct this situation, and the question remains as to how best to improve RT compliance.

## Conflict of Interest

None declared.
